# Mitochondria-Targeted Biophysical Priming of Autologous Biologics for Skin Regeneration and Wound Repair

**DOI:** 10.3390/ijms27052201

**Published:** 2026-02-26

**Authors:** Geun-Ho Kang, Kilyong Lee, Chang Hwan Jeon, Seong Kyoung Kim, SungHoon Cho

**Affiliations:** 1 Spine Center, Seoul Yes Hospital, 2736, Yonggu-daero, Suji-gu, Yongin-si 16868, Gyeonggi-do, Republic of Korea; yeskylee2@gmail.com (K.L.); cerenadecico@naver.com (C.H.J.); nk8275@naver.com (S.C.); 2 R&D Center, SeoulYesBio Co., Ltd., 2746, Yonggu-daero, Suji-gu, Yongin-si 16868, Gyeonggi-do, Republic of Korea; rlatjdrud425@naver.com

**Keywords:** mitochondria, skin aging, chronic wounds, platelet-rich plasma (PRP), stromal vascular fraction (SVF), bone marrow aspirate concentrate (BMAC), mesenchymal stromal cells (MSCs), photobiomodulation (PBM), low-intensity pulsed ultrasound (LIPUS), biophysical priming

## Abstract

Skin aging, photoaging, and chronic wounds are increasingly recognized to be driven by mitochondria-centered mechanisms characterized by oxidative stress, defective mitophagy, and impaired bioenergetics in cutaneous cells. Autologous biologics, including platelet-rich plasma, stromal vascular fraction, bone marrow aspirate concentrate, and mesenchymal stromal/stem cell–derived products, are widely used for skin rejuvenation and wound repair. Recent studies have suggested that many of these effects are mediated by mitochondrial mechanisms, including metabolic reprogramming, redox modulation, and intercellular mitochondrial transfer. Concurrently, biophysical modalities such as red/near-infrared photobiomodulation (PBM), low-intensity pulsed ultrasound, mechanical stimulation, and nanoengineered cues can modulate mitochondrial function in skin-relevant cells. In this review, we integrate these lines of evidence to introduce the concept of mitochondria-targeted biophysical priming of autologous biologics for dermatological applications. We summarize the mitochondrial biology in skin pathology, evaluate these biologics as mitochondria-active therapies, and outline ex vivo priming implementation using PBM, ultrasound, or mechanical stimulation. Finally, we discuss key regulatory considerations that support clinical translation.

## 1. Introduction

The skin is continuously exposed to various environmental and physiological stressors, including ultraviolet (UV) radiation, air pollution, mechanical stress, metabolic perturbations, and tissue injuries associated with wounds or surgical procedures. These stressors converge on the mitochondria, inducing oxidative damage to the mitochondrial DNA (mtDNA), impaired oxidative phosphorylation, excessive production of reactive oxygen species (ROS), and defective mitophagy. Consequently, mitochondrial dysfunction has emerged as a hallmark of photoaging, intrinsic aging, as well as inflammatory and fibrotic skin disorders [1,2,3,4]. In chronic wounds, sustained mitochondrial dysfunction and oxidative stress in keratinocytes, fibroblasts, and immune cells contribute to cellular senescence, reduced migratory capacity, and persistent inflammation [5,6,7,8].

Mitochondria act as central metabolic hubs governing skin homeostasis and participate in essential processes ranging from keratinocyte differentiation and epidermal barrier formation to extracellular matrix (ECM) synthesis by dermal fibroblasts [9]. The high bioenergetic demands of skin tissue regeneration and the complex phases of wound healing (inflammation, proliferation, and remodeling) depend heavily on mitochondrial metabolic flexibility and ATP production [8]. In addition to bioenergetics, mitochondria regulate intracellular calcium homeostasis, apoptosis, and the signaling pathways that govern cell survival and senescence [10]. Therefore, therapeutic strategies that modulate mitochondrial quality-control mechanisms (fission, fusion, and mitophagy) or enhance mitochondrial bioenergetics have the potential to reverse skin aging and accelerate tissue regeneration [11]. Restoring mitochondrial function entails not only increasing energy production but also reestablishing the signaling integrity necessary for tissue repair.

Autologous human-derived biological materials, including platelet-rich plasma (PRP), bone marrow aspirate concentrate (BMAC), and stromal vascular fraction (SVF), have become increasingly important in regenerative dermatology and aesthetic medicine. PRP, platelet-poor plasma (PPP), and related plasma preparations are widely used for facial rejuvenation, acne and surgical scar improvement, and hair growth promotion [12,13,14]. Mesenchymal stromal/stem cell (MSC)-based therapies, including bone marrow–derived mesenchymal stem cells, adipose-derived stem cells, and their conditioned media or exosomes, have been shown to improve acute and chronic wound healing and treat radiation-induced skin injury [15,16,17]. These approaches offer significant advantages by leveraging endogenous repair mechanisms and obviating the need for allogeneic transplantation or gene therapy. However, both the clinical and mechanistic literature remains heterogeneous, with substantial variability in product preparation, dosing, and outcome measures. Moreover, mitochondria-centered explanations are supported mainly by associative or preclinical data; mitochondrial endpoints show variable and sometimes inconclusive changes across models, and causal links to clinical outcomes remain to be established [18]. Accordingly, throughout this review we treat mitochondrial mechanisms as plausible but not definitive and discuss them alongside alternative, non-mitochondrial mechanisms (e.g., growth-factor signaling, immunomodulation, angiogenesis, and ECM remodeling).

Throughout this review, we use the term “autologous biologics” to refer to minimally manipulated processed blood- and human-tissue-derived preparations obtained from the same individual, including PRP, PPP, BMAC, and SVF. Despite variations in cellular composition, platelet and leukocyte content, stromal cell fraction, and processing methods, these preparations share two key characteristics: (i) an autologous origin that minimizes immunogenicity and infection risk and (ii) a regulatory status that is generally consistent with minimal manipulation, which makes them suitable for office-based procedures in dermatology and wound care without the infrastructure required for extensive cell expansion or genetically modified therapies [13,19,20,21].

For the purposes of this review, “biophysical priming” is defined as the transient exposure of autologous biologics to controlled physical cues (e.g., photobiomodulation (PBM), low-intensity pulsed ultrasound (LIPUS), and mechanical forces) within a closed system, explicitly excluding the addition of exogenous growth factors or agents that promote cell proliferation [22,23]. Clinically, physical modalities, such as red/near-infrared (NIR) PBM, therapeutic ultrasound, mechanical stretching, and radiofrequency, are widely used for skin rejuvenation and scar management. Accumulating evidence indicates that their therapeutic effects are largely mediated by mitochondrial photoreceptors, mechanosensitive pathways, and redox signaling [24,25,26,27]. This convergence of findings raises a pivotal question: rather than applying physical energy solely to the skin surface, can we biophysically “prime” autologous biologics (e.g., PRP, BMAC, SVF) at the mitochondrial level prior to their clinical application? Therefore, this review aimed to (1) summarize the role of mitochondria in skin health, aging, and wound repair; (2) characterize PRP-, BMAC-, and SVF-based interventions as mitochondria-modulating therapies; (3) review biophysical modulators of mitochondrial function in skin-relevant cells; and (4) propose conceptual designs for mitochondria-targeted biophysical priming of human-derived biological materials for skin rejuvenation and wound healing.

## 2. Mitochondria in Skin Health, Aging, and Wound Repair

### 2.1. Mitochondrial Mechanisms in Skin Aging and Photoaging

Mitochondria regulate energy production, ROS generation, apoptosis, and innate immune signaling in keratinocytes, melanocytes, dermal fibroblasts, and skin stem cells. Chronic UV exposure causes cumulative mtDNA damage in both the epidermal and dermal compartments. Large-scale mtDNA deletions and point mutations are characteristic of photoaged skin and contribute to reduced capacity and increased oxidative stress [2,4]. Mitochondrial ROS activate matrix metalloproteinases, degrade dermal collagen and elastin, and promote the production of pro-inflammatory cytokines, driving wrinkle formation and loss of dermal elasticity [1,3].

At the cellular level, oxidative stress in keratinocytes and fibroblasts activates damage-response pathways (e.g., p53, NF-κB), alters cytokine release, and accelerates cellular senescence [3,4]. Dermal fibroblast autophagy and proliferation are strongly linked to skin anti-aging, and dysregulated autophagy contributes to the accumulation of damaged mitochondria and dysfunctional ECM remodeling [1,28]. Thus, strategies for preserving or restoring mitochondrial function in skin cells are of central interest for antiaging and rejuvenation.

### 2.2. Mitochondria in Wound Healing and Chronic Wounds

Acute wound healing progresses through several stages, including hemostasis, inflammation, proliferation, and remodeling, with mitochondria playing a key role at each stage. During the acute inflammatory response, macrophages undergo metabolic reprogramming from glycolytic, pro-inflammatory states toward oxidative, pro-resolving phenotypes. Mitochondrial respiration and controlled ROS production are essential to restore tissue homeostasis [6,8].

In chronic wounds, persistent oxidative stress, mitochondrial dysfunction, and defective mitophagy in keratinocytes and fibroblasts lead to senescence, reduced proliferation, and decreased migration [4,5,7]. Mitochondrial components released from damaged cells and persistent activation of inflammatory pathways, both tightly linked to mitochondrial dysfunction, contribute to a non-resolving inflammatory environment [6]. Given their central role, mitochondria have emerged as attractive therapeutic targets for wound healing, with strategies ranging from antioxidant and metabolic regulators to mitochondrial transplantation [4,18]. Therefore, autologous biological agents that can repair mitochondrial damage, normalize ROS levels, and promote mitochondrial biogenesis in skin cells may have better wound-healing efficacy than pharmacological agents or growth factors. 

### 2.3. Mitochondria in Mesenchymal Stromal/Stem Cell-Mediated Cutaneous Repair

For MSCs, mitochondrial dynamics, mitophagy, and mitochondrial ROS generation are key determinants of MSC fate, influencing their resistance to apoptosis, immunomodulatory phenotype, and secretome composition, including conditioned media and extracellular vesicles (EVs) [15,17,18]. In cutaneous wound models, MSCs with preserved mitochondrial function show improved survival and enhanced support for re-epithelialization, angiogenesis, and ECM remodeling, whereas mitochondrial dysfunction is associated with impaired engraftment and reduced regenerative efficacy [15,18].

**Figure 1:**
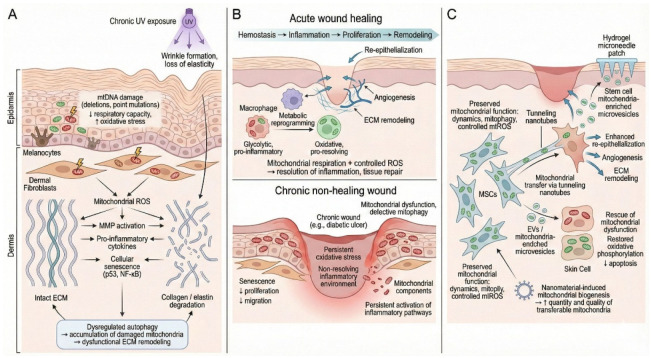
Central role of mitochondrial function in skin homeostasis, aging, wound healing, and regenerative therapies. (**A**) Mitochondria in skin aging and photoaging. (**B**) Mitochondria in acute vs. chronic wounds. (**C**) Mitochondria in MSCs relevant to cutaneous repair.

In addition to their intrinsic metabolic state, MSCs can serve as mitochondrial donors to stressed or damaged cells. However, as cautioned in [18], the magnitude, reproducibility, and functional relevance of such transfer are highly context-dependent, and the available evidence remains largely limited to preclinical models. Consequently, mitochondrial transfer should be regarded as one of several plausible paracrine and immunomodulatory mechanisms [18,29,30,31]. From a translational perspective, hydrogel microneedles delivering mitochondria-enriched microvesicles derived from stem cells have been reported to accelerate healing in radiation-compromised wounds, partly by restoring oxidative phosphorylation and mitigating apoptosis [30]. In addition, nanomaterial-induced mitochondrial biogenesis in MSCs may augment both the yield and quality of transferable mitochondria, thereby improving transfer efficiency [31,32]. As summarized in Figure 1, these collective findings suggest that modulating MSC mitochondrial quality provides a rationale for biophysical priming. Nevertheless, clinical translation remains unproven and requires rigorous validation.

## 3. Human-Derived Biological Materials for Skin Rejuvenation and Their Mitochondrial Effects

### 3.1. Clinical and Cellular Effects of Platelet-Rich Plasma and Platelet-Poor Plasma in the Skin

PRP is prepared by centrifuging autologous whole blood to concentrate platelets and, variably, leukocytes and plasma proteins. In dermatology, PRP is used for facial rejuvenation, melasma, atrophic acne scars, surgical scars, and hair restoration, often as an adjunct to laser therapy or as a microneedle. Clinical and histological studies indicate that intradermal PRP injections increase dermal thickness, collagen and elastin content, and vascularity, with improvements in skin texture, fine wrinkles, and pigmentation [12,13,14,33].

PRP and PPP have been shown to enhance dermal fibroblast proliferation, collagen synthesis, and expression of ECM-related genes in vitro. Previous studies demonstrated that autologous PRP and PPP stimulate human dermal fibroblast proliferation and upregulate type I collagen expression, supporting their use as adjunct therapies for laser-based rejuvenation [33]. PRP-derived growth factors (e.g., PDGF, TGF-β, VEGF) and cytokines activate fibroblasts and endogenous precursor cells, thereby promoting ECM remodeling and angiogenesis [13,14].

PRP has been evaluated for cutaneous wound healing in patients with diabetic ulcers and complex surgical wounds. Recent reports have indicated that PRP promotes diabetic wound healing by regulating mitochondrial transport, modulating ROS-dependent epithelial–mesenchymal transition (EMT), and restoring redox homeostasis. These findings imply that mitochondria-mediated mechanisms underlie the efficacy of PRP compared with single growth factor therapy [29,34].

### 3.2. Mitochondrial Modulation by Platelet-Rich Plasma in Skin Cells

Several studies have demonstrated the direct and indirect mitochondrial effects of PRP on dermal and epidermal cells. In human dermal fibroblasts, an “enhanced PRP” (ePRP) formulation was shown to significantly modulate oxidative phosphorylation and glycolysis. Specifically, the experimental results demonstrated that ePRP induces metabolic reprogramming in fibroblasts, which is characterized by a shift toward glycolysis to fuel rapid proliferation. These metabolic shifts have been mechanistically linked to enhanced wound closure, both in vitro and in vivo [13,35].

Furthermore, in human keratinocytes exposed to environmental stress, specifically UVB radiation, treatment with PRP demonstrated protective effects by substantially reducing markers of oxidative and endoplasmic reticulum stress, including GRP78, CHOP, and ATF4. The reduction in these stress markers and consequent decrease in cell death are associated with enhanced mitochondrial stress–handling capacity and diminished ROS-mediated cell damage [36,37,38].

Recent research has suggested a broader role for PRP in regulating the complex mitochondrial microenvironment in chronic wounds. In diabetic wound models, PRP has been hypothesized to attenuate mitochondrial dysfunction and oxidative stress across diverse cell populations, including keratinocytes, endothelial cells, and immune cells, thereby normalizing redox signaling and promoting appropriate epithelial and endothelial transitions necessary for tissue repair [7,34].

Collectively, these studies suggest that PRP may modulate mitochondrial and redox endpoints in dermal and epidermal cells under specific experimental conditions, in addition to its established role in delivering growth factors and cytokines [13,35,36,37,38]. However, existing data are highly heterogeneous and derived predominantly from in vitro or preclinical models. Consequently, the true magnitude and clinical translation of these mitochondria-related effects are likely contingent upon PRP formulation, dosing parameters, and the target microenvironment. Therefore, mitochondrial modulation should be viewed as a plausible, but not exclusive, component of PRP. Importantly, PRP outcomes in dermatologic indications are influenced by preparation and activation protocols (e.g., platelet/leukocyte content), dosing frequency, and concomitant procedures, contributing to variable effect sizes across studies [12,13,14]. In addition, PRP-mediated tissue responses are multifactorial and may arise from growth-factor signaling, inflammatory regulation, angiogenesis, and ECM remodeling in addition to putative mitochondrial effects. Accordingly, this review does not position PRP as superior to MSC-based approaches, but discusses PRP and MSC/BMAC modalities as potentially complementary options whose efficacy and dominant mechanisms depend on indication and study design.

### 3.3. Mesenchymal Stromal/Stem Cell-Based Therapies and Bone Marrow Aspirate Concentrate for Skin

MSCs derived from bone marrow and adipose tissue have been extensively explored for cutaneous wound repair. However, despite encouraging experimental and early clinical reports, MSC-based interventions for cutaneous indications face substantial limitations. These include heterogeneity in cell sourcing and processing, inconsistent or non-standardized potency assays, variable in vivo retention and survival after delivery, and uncertainty regarding the optimal dose, timing, and route of administration. Furthermore, batch-to-batch variability in secretome and extracellular vesicle (EV) composition, coupled with practical and regulatory constraints, can further contribute to variable and sometimes inconclusive clinical outcomes [15,16,17]. Currently, for damage induced by complex or multiple external stressors, attention is increasingly focused on minimally processed autologous biologics (e.g., BMAC) as well as cell-free approaches like MSC-derived secretomes [17,18,30].

BMAC offer a minimally processed and clinically accessible source of MSCs, hematopoietic progenitors, and immune cells. Although primarily studied in orthopedics, BMAC has been increasingly applied to complex wounds and radiation-associated skin injuries and frequently combined with scaffolds or hydrogels to enhance retention. Recent studies have demonstrated that the efficacy of MSC-based therapies is mediated by the restoration of tissue bioenergetics in the recipient skin cells. Specifically, studies have indicated that the heterogeneous cell population in BMAC, particularly endothelial progenitor cells along with MSCs, promotes robust neovascularization and immunomodulation. This physiological restoration re-establishes the oxygen and nutrient supply critical for mitochondrial oxidative phosphorylation in hypoxic wound environments [39,40].

**Figure 2:**
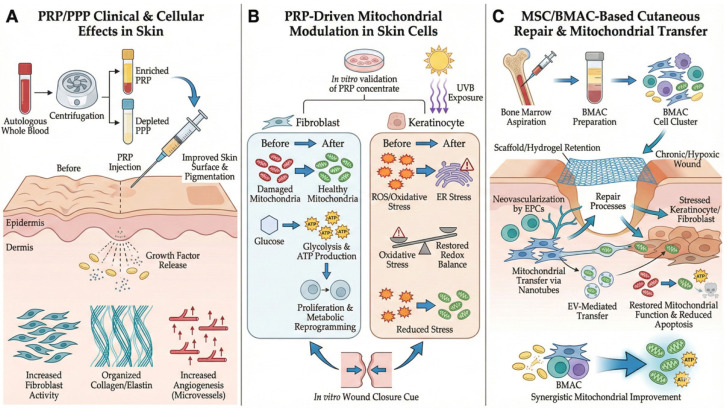
Mechanisms of action for platelet-rich plasma (PRP) and mesenchymal stromal/stem cell (MSC)-based therapies in cutaneous regeneration, focusing on mitochondrial modulation. (**A**) Clinical and cellular effects of PRP and plasma-derived preparations in the skin. (**B**) PRP-driven mitochondrial modulation and stress response in skin cells. (**C**) MSC-based therapies and BMAC: mitochondrial transfer and metabolic coupling in cutaneous repair.

One proposed mechanism underlying the benefits of MSCs and BMAC is the restoration of cellular bioenergetics in recipient skin cells, as summarized in Figure 2. This includes mitochondrial support via tunneling nanotubes or the EV-mediated delivery of mitochondrial components, processes that reportedly enhance mitochondrial respiration, ATP production, and cell survival under oxidative or physiological stress [18,30]. However, the relative contribution of mitochondrial transfer compared to other paracrine and immunomodulatory pathways remains incompletely defined and is highly context-dependent. Furthermore, platelet-rich components may potentiate these regenerative processes; for instance, the combined application of PRP and BMAC has been shown to augment metabolic reprogramming and mitochondrial functional indices in specific models [8,29]. Accordingly, mitochondria-oriented priming is presented here as a testable therapeutic strategy rather than an established determinant of clinical efficacy.

## 4. Nanoengineered Materials and Surfaces for Mitochondrial Modulation

### 4.1. Photobiomodulation in Dermal Fibroblasts and Cutaneous Repair

PBM involves the use of low-intensity red and NIR light delivered by lasers or light-emitting diodes (LEDs) including organic LEDs (OLEDs). Treatment typically utilizes wavelengths in the 600–900 nm range, at fluences of ~0.5–10 J/cm^2^. Cytochrome c oxidase and other mitochondrial chromophores absorb these wavelengths, leading to increased electron-transport-chain activity, enhanced ATP synthesis, and modulation of ROS and nitric oxide signaling [32,41].

Several studies have shown that PBM delivered within defined therapeutic ranges enhances human dermal fibroblast proliferation and migration in vitro [24,31,42], while also promoting collagen type I and III synthesis and reorganizing the ECM [14,31]. Furthermore, PBM enhances mitochondrial function by elevating mitochondrial membrane potential and ATP production while concurrently mitigating excessive ROS generation and apoptosis under oxidative stress conditions [24,31]. A study investigating the effects of PBM on dermal fibroblasts demonstrated significant mitochondrial enhancement and cell proliferation within specific power density and fluence ranges, whereas parameters outside these windows showed no effect or were even inhibitory [24,42].

Clinically, PBM has demonstrated beneficial effects on skin rejuvenation by reducing fine wrinkles, improving texture and pigmentation, and enhancing wound healing, particularly when used as an adjunctive therapy [12,16,27]. A recent study using a OLED-based PBM showed that phototherapy improves mitochondrial function, attenuates oxidative stress, and stimulates cytoprotective factor production in skin cells. These findings highlight the role of mitochondria-mediated mechanisms in vivo [43].

Based on these results, recent in vitro studies have investigated the combination of PBM with PRP and plasma-rich growth factors (PRGFs) in human dermal fibroblasts. The combination of laser PBM and PRP/PRGF resulted in additive or synergistic increases in fibroblast proliferation and mitochondrial activity compared with either modality alone. These results suggest that PBM can potentiate the mitochondrial and regenerative effects of platelet concentrates [44], providing a proof-of-concept for PBM-primed plasma therapies for skin rejuvenation.

### 4.2. Ultrasound and Mechanical Stimulation

Ultrasound is a non-invasive mechanical stimulus widely used in musculoskeletal rehabilitation and is increasingly used in aesthetic dermatology. High-intensity focused ultrasound (HIFU) is clinically used for skin tightening, and histological analyses have shown increased dermal collagen and elastin levels following HIFU. These effects appear to be mediated, at least in part, by the modulation of caveolin-1 and downregulation of p53 activity, pathways that intersect with mitochondrial stress responses and fibroblast activation [1,25].

By contrast, LIPUS and other therapeutic ultrasound regimens have been reported to increase the proliferation of human skin fibroblasts by activating the MAPK/ERK and PI3K–Akt pathways [45,46]. These modalities have also been shown to enhance collagen and glycosaminoglycan production and promote matrix contraction in fibroblast-populated hydrogels [47,48]. In addition, ultrasound can modify the mechanical and biological properties of ligaments and other soft-tissue fibroblasts, including cytoskeletal reorganization and increased ECM synthesis [49].

Although the direct measurement of mitochondrial endpoints in ultrasound-treated skin fibroblasts remains limited, studies on other cell types have indicated relevant bioenergetic mechanisms. Specifically, LIPUS has been reported to restore the mitochondrial membrane potential, reduce mitochondrial ROS, and promote mitophagy and mitochondrial biogenesis, raising the possibility that analogous responses may occur in skin cells [4,8,50].

Mechanical stretching is another important regulator of skin biology. Soft-tissue expansion during reconstructive surgery relies on chronic mechanical stretching to induce de novo skin formation. Mechanistic studies have indicated that mechanical stretching activates multiple signaling pathways in keratinocytes, dermal fibroblasts, and MSCs, thereby altering gene-expression profiles, proliferation, migration, and ECM remodeling [26,51]. Stretch-induced fibroblast activation has been linked to mechanosensitive channels (e.g., Piezo1) and YAP/TAZ signaling, which interact with mitochondrial dynamics and redox regulation [4,52].

Collectively, these findings support the broader concept that mechanical and ultrasonic stimuli can reprogram fibroblast and stem cell functions, plausibly through mitochondrial mechanotransduction and redox pathways. Therefore, incorporating such stimuli into ex vivo processing workflows for human-derived biologics may provide a controllable strategy for mitochondrial priming in skin applications.

### 4.3. Nanoengineered and Nanoelectronic Approaches

Nanomaterials with defined electronic and structural properties are increasingly used to modulate mitochondrial function. For example, engineered MoS_2_ nanoflowers have been reported to stimulate mitochondrial biogenesis via the upregulation of PGC-1α and TFAM, increase mtDNA copy number and respiratory chain protein expression, and enhance mitochondrial respiratory capacity and ATP production across multiple mammalian cell types, including human MSCs [41]. Similarly, other low-dimensional nanomaterials, such as graphene oxide, have been shown to modulate mitochondrial bioenergetics and metabolic switching in stem cells [53].

**Figure 3:**
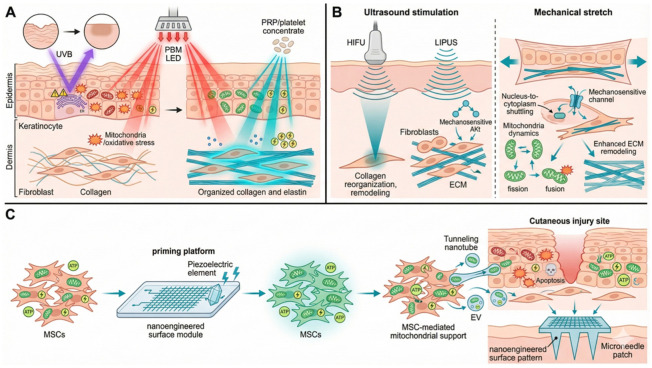
Strategies for cutaneous regeneration and mitochondrial modulation using biophysical stimuli and nanoengineered stem cell therapies. (**A**) Synergistic effects of photobiomodulation (PBM) and platelet-rich plasma (PRP) on skin rejuvenation. (**B**) Mechanotransduction pathways activated by ultrasound and mechanical stretching. (**C**) Nanoengineered priming of MSCs for enhanced mitochondrial transfer in wound repair.

Recent work has demonstrated that MoS_2_ nanoflowers can convert MSCs into “mitochondrial biofactories,” thereby increasing both the abundance and functional quality of mitochondria available for intercellular transfer and improving MSC-mediated rescue of mitochondrial dysfunction in stressed recipient cells [41]. For clinical skin applications, nanoengineered surfaces such as conductive or piezoelectric coatings integrated into microneedle patches or ex vivo processing platforms may represent a more translatable strategy than the direct incorporation of free nanoparticles. As shown in Figure 3, surface-based approaches can deliver controlled electrical and/or mechanical cues that promote mitochondrial biogenesis and functional conditioning during ex vivo processing, thereby enhancing mitochondrial support provided by MSCs to injured cutaneous cells.

## 5. Mitochondria-Targeted Biophysical Priming for Skin Improvement

### 5.1. Definition and Basis of Biophysical Priming

Synthesizing the evidence summarized above, this conceptual framework includes three main propositions: (1) mitochondrial dysfunction is a key driver of skin aging, photoaging, and chronic wounds [2,7]; (2) therapies based on PRP, BMAC, SVF, and MSCs mediate some of their cutaneous benefits by modulating mitochondrial function, oxidative stress, and intercellular mitochondrial transfer [33,35]; and (3) PBM, ultrasound, mechanical stimulation, and nanoengineered cues provide non-pharmacological means to enhance mitochondrial quality control in fibroblasts, keratinocytes, and MSCs [31,42]. Biophysical priming is defined as a brief ex vivo exposure of human-derived biological materials, such as PRP, BMAC, and SVF, to control physical stimuli in a closed system, with the explicit aim of optimizing mitochondrial function and redox balance prior to skin application.

### 5.2. Photobiomodulation-Primed Platelet-Rich Plasma for Photoaged Facial Skin as an Example Priming Scenario

The following priming scenarios serve as illustrative, hypothesis-generating examples designed to evaluate feasibility within closed-system workflows. They do not constitute clinical recommendations and mandate rigorous prospective validation. In the proposed protocol, PRP was prepared from autologous whole blood using standard centrifugation procedures. Without breaching the sterile seal, the PRP is then subjected to PBM by irradiating the sealed syringe or bag with low-intensity red-to-NIR light (660–830 nm) at a fluence of approximately 0.5–3 J/cm^2^. This closed-system approach was designed to minimize contamination risks and processing steps while enabling controlled biophysical stimulation.

Several studies have demonstrated that NIR low-level light can preserve mitochondrial function, ATP production, and viability of platelet concentrates during storage, while limiting lactate generation and oxidative stress [54,55]. Based on this rationale, this ex vivo priming step was hypothesized to preserve or modestly increase the platelet mitochondrial membrane potential and ATP content, maintain ROS signaling within a non-damaging range, reduce markers of platelet activation and apoptosis, and shift the profile of platelet-derived EVs toward a more cytoprotective composition.

PBM-primed PRP was administered to the facial skin via intradermal injection, optionally in combination with microneedling. Additional PBM may be applied as an adjunct to further enhance cutaneous rejuvenation, in line with established LED-based skin-rejuvenation protocols [56]. This integrated strategy conceptually couples mitochondria-targeted biophysical conditioning of PRP with conventional aesthetic delivery techniques to improve the efficacy and durability of clinical outcomes in photoaged skin.

### 5.3. Ultrasonically or Mechanically Primed Bone Marrow Aspirate Concentrate for Chronic Wounds

As above, this scenario is conceptual and intended to guide future testing rather than to prescribe clinical practice. In this protocol, autologous BMAC was first prepared under standard aseptic conditions and release criteria for the base products. The resulting preparation was then biophysically primed ex vivo by either LIPUS—for example, 1 MHz with spatial-average intensities of ~20–30 mW/cm^2^ delivered for 5–10 min in pulsed mode—or nanovibrational stimulation at ~1 kHz with nanometer-scale amplitudes. These parameter ranges are based on preclinical studies demonstrating that such stimuli modulate mesenchymal stem cell chondrogenesis, autophagy, and mechanotransduction-linked metabolic reprogramming. Based on the findings that LIPUS promotes chondrogenesis of mesenchymal stem cells via autophagy regulation and that nanovibrational stimulation (1 kHz, 30–90 nm amplitude) activates energy-demanding metabolic programs and induces controlled ROS, this priming strategy is hypothesized to increase mitochondrial biogenesis and respiratory capacity within MSCs. This approach aims to enrich mitochondrial content and function in secreted EVs and shift the overall secretome toward a more pro-resolving, pro-regenerative profile. Such primed secretome products would then be suitable for subsequent delivery to cutaneous lesions via topical hydrogels, microneedle patches, medical device, or perilesional injections around chronic wound margins [57,58,59].

### 5.4. Minimal Manipulation and Safety Considerations

From a regulatory perspective, the development of priming strategies for autologous human-derived preparations (e.g., PRP, BMAC, SVF) must explicitly consider the “minimal manipulation” thresholds defined for human cell- and tissue-based products in the United States, Europe, Japan, China, and South Korea. Within these regulatory frameworks, procedures that preserve relevant biological properties are typically categorized as minimal manipulations. Conversely, substantial modifications necessitate classification into biological drugs or advanced therapy medicinal products that require rigorous premarket authorization [60,61]. Accordingly, brief, non-thermal photobiomodulation, physical stimulation, or low-intensity ultrasound applied to sealed containers without additives or ex vivo culture is more likely to be deemed minimal manipulation, provided that the induced phenotypic changes are modest and reversible. By contrast, long-term exposure to nanomaterials, strong electric fields, or device-mediated conditioning that drives durable reprogramming is usually considered to be too vigorous to qualify as minimal manipulation and is subject to heightened regulatory scrutiny [62,63]. To advance clinical applications under these regulatory guidelines, preclinical studies should quantitatively characterize mitochondrial endpoints (e.g., mitochondrial membrane potential, ATP content, mitochondrial ROS, mtDNA copy number, mitophagy/biogenesis markers) and link them to skin-relevant functional readouts (e.g., dermal fibroblast proliferation and migration, collagen and elastin synthesis, three-dimensional skin-equivalent remodeling, in vivo wound repair, photoaging models). Concurrently, nonclinical safety testing in line with current cellular therapy guidelines must confirm the absence of unintended activation, prothrombotic or inflammatory responses, and genotoxicity [64,65].

## 6. Conclusions

**Figure 4:**
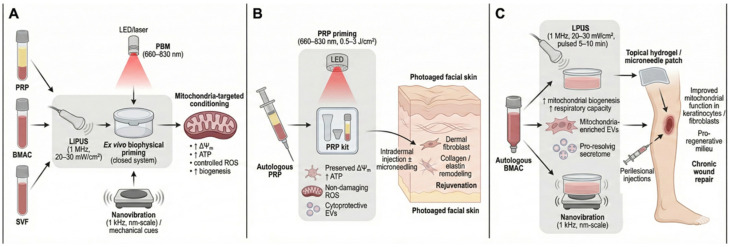
Mitochondria-targeted biophysical priming for skin improvement. (**A**) Conceptual framework for the mitochondria-targeted biophysical priming of autologous biologics in a closed system. (**B**) PBM-primed PRP for photoaged facial skin. Following standard PRP preparation, the sealed syringe or kit is irradiated with red/NIR light without compromising sterility. (**C**) Application scenario demonstrating ultrasound- or mechanically primed BMAC for chronic wounds. Autologous BMAC is primed ex vivo using LIPUS or nanovibration to enhance mitochondrial biogenesis and respiratory capacity in MSC-enriched fractions, thereby enriching EV/secretome outputs with pro-resolving and pro-regenerative activity.

This review proposes a mitochondria-targeted biophysical priming framework for autologous biologics in skin regeneration and wound repair. As discussed, mitochondrial dysfunction is implicated in skin aging and chronic wound pathology [2,66,67,68]. While PRP, BMAC, SVF, and autologously derived MSC-based approaches may modulate tissue bioenergetics, the reported effects are variable and their mechanistic attribution remains uncertain. Accordingly, we present the priming concept primarily as hypothesis-generating in Figure 4. Short-term ex vivo biophysical cues (e.g., PBM, ultrasound, mechanical stimulation, electromagnetic fields, and nanovibration) may affect mitochondria-related endpoints; however, rigorous standardization, transparent reporting of negative/neutral results, and adequately powered studies are required before prescriptive clinical recommendations can be made.

The clinical feasibility of this strategy lies in its alignment with minimal-manipulation regulatory frameworks. Unlike genetic modification or extensive culturing, biophysical priming in closed systems offers a compliant pathway to enhance therapeutic potency without compromising safety or triggering complex regulatory classifications [29,68,69,70]. The proposed protocols—PBM-primed PRP for cosmetic enhancement and mechanically conditioned MSCs for refractory wounds—are immediate translational applications of this concept.

Rigorous oversight regarding standardization, safety, and stability is required to apply products derived from these concepts in clinical settings. Future research must prioritize the following: (1) defining precise dose–response windows; (2) establishing rapid, point-of-care assays for quality control (e.g., mitochondrial membrane potential, metabolic flux) to validate priming efficacy; and (3) mapping the optimal temporal sequencing between ex vivo priming and in vivo application. Ultimately, integrating these biophysically primed biologics with emerging precision medicine approaches will enable clinicians to deliver customized interventions, thereby shifting the paradigm from generalized repair to targeted cellular regeneration.

## Data Availability

No new data were created or analyzed in this study. Data sharing is not applicable to this article.
